# Identifying Genes Involved in Cyclic Processes by Combining Gene Expression Analysis and Prior Knowledge

**DOI:** 10.1155/2009/683463

**Published:** 2009-03-03

**Authors:** Wentao Zhao, Erchin Serpedin, Edward R Dougherty

**Affiliations:** 1 Department of Electrical and Computer Engineering, Texas A&M University, College Station, TX 77843-3128, USA; 2 Computational Biology Division, Translational Genomics Research Institute, 400 North Fifth Street, Suite 1600, Phoenix, AZ 85004, USA

## Abstract

Based on time series gene expressions, cyclic genes can be recognized via spectral analysis and statistical periodicity detection tests. These cyclic genes are usually associated with cyclic biological processes, for example, cell cycle and circadian rhythm. The power of a scheme is practically measured by comparing the detected periodically expressed genes with experimentally verified genes participating in a cyclic process. However, in the above mentioned procedure the valuable prior knowledge only serves as an evaluation benchmark, and it is not fully exploited in the implementation of the algorithm. In addition, partial data sets are also disregarded due to their nonstationarity. This paper proposes a novel algorithm to identify cyclic-process-involved genes by integrating the prior knowledge with the gene expression analysis. The proposed algorithm is applied on data sets corresponding to *Saccharomyces cerevisiae* and *Drosophila melanogaster*, respectively. Biological evidences are found to validate the roles of the discovered genes in cell cycle and circadian rhythm. Dendrograms are presented to cluster the identified genes and to reveal expression patterns. It is corroborated that the proposed novel identification scheme provides a valuable technique for unveiling pathways related to cyclic processes.

## 1. Introduction

The eukaryotic cell hosts several cyclic molecular processes, for example, cell cycle and circadian rhythm. The transcriptional events in these processes can be quantitatively observed by measuring the concentration of the messenger RNA (mRNA), which is transcribed from DNA and serves as the template for synthesizing the corresponding protein. To achieve this goal, the microarray experiments exploit high-throughput gene chips to snapshot genome-wide gene expressions sequentially at discrete time points. The sampled time series data present three main characteristics. First, most data sets present small sample size, for example, no more than 50 data points. Obtaining large sample size data sets is not financially affordable, and besides, in the long run the cell culture loses synchronization and the data become meaningless if they are sampled much later on. Second, the data might not be evenly sampled, and many time points could be missing. In order to capture critical events with minimal cost, biologists usually conduct microarray experiments and make measurements when these events happen. Third, the data are highly corrupted by experimental noise, and a robust stochastic analysis is desired.

Based on time series data, various approaches have been proposed to identify periodically expressed genes, which are sometimes believed to be involved in the cell cycle. Assuming the cell cycle signal to be a simple sinusoid, Spellman et al. [1] and Whitfield et al. [2] performed Fourier transformations on the data sampled with different synchronization methods, Wichert et al. [3] applied the traditional periodogram and Fisher's test, while Ahdesmäki et al. [4] implemented a robust periodicity test assuming non-Gaussian noise. In [5], Giurcǎneanu explored the stochastic complexity of detecting periodically expressed genes by means of generalized Gaussian distributions. Alternatively, Luan and Li [6] employed guide genes and constructed cubic B-spline-based periodic functions for modeling, while Lu et al. [7] employed up to third harmonics to fit the data and proposed a periodic normal mixture model. De Lichtenberg et al. [8] compared the approaches [1, 6, 7] and proposed a new score combining the periodicity and regulation magnitude. Interestingly, the mathematically more advanced methods seem not to achieve a better performance compared with the original Spellman's method that relies on the Fast Fourier Transform (FFT) method. As an important observation, notice that the majority of these works deal only with evenly sampled data. When data points are missing, in general for the adopted methods, the vacancies are usually filled by interpolation in time domain for all genes, or the genes are disregarded if there are more than 30% of data samples missing.

The biological experiments generally output unevenly spaced measurements. The change of sampling frequency can be attributed to missing data. Besides, the measurements are usually event-driven, that is, more observations are recorded when certain biological events happen, and the observational process is slowed down when the cell remains quiet or no event of interest occurs. Therefore, the analysis based on unevenly sampled data sets is practically more desirable and technically more challenging. Notice that in the case of uneven sampling, the harmonics exploited in the discrete Fourier transform (DFT) are no longer orthogonal. Lomb [9] and Scargle [10] demonstrated that a phase shift suffices to make the sine and cosine terms orthogonal again, and consequently a spectral estimator can be designed in the presence of uneven sampling. The Lomb-Scargle scheme has been exploited by Glynn et al. [11] in analyzing the budding yeast data set. Notice also that a number of alternative schemes were proposed recently to cope with missing and/or irregularly spaced data samples. Stoica and Sandgren [12] updated the traditional Capon method to cope with the irregularly sampled data. Wang et al. [13] designed the missing-data amplitude and phase estimation (MAPES) approach, which estimated the missing data and spectra iteratively through the Expectation Maximization (EM) algorithm. Although Capon and MAPES methods aim to achieve a better spectral resolution than Lomb-Scargle periodogram, for small sample size, the simpler Lomb-Scargle scheme appears to possess better performance in the presence of realistic biological data [14].

Most of the algorithms proposed in literature identify cyclic genes by exploiting mathematical models to explain the gene's time series pattern. Employing these models and statistical tests, the periodically expressed genes are normally identified. Finally, the detected genes are compared with the genes that had been experimentally discovered to participate in specific processes like cell cycle. Notice that these practically verified cycle-involved genes only serve as a golden benchmark to evaluate the performance of the proposed identification algorithms. They are not fully exploited in the implementation of the identification algorithm. Notice also that most of the existing algorithms fail to utilize all the available data information. For example, the elutriation data provided in [1] was usually discarded when performing the spectral analysis. In other experiments, some data sets were also disregarded due to either loss of synchronization or nonstationarity. Herein, we propose a novel algorithm to detect periodically expressed genes by integrating the gene expression analysis with the valuable prior knowledge offered by all available data. The prior knowledge can consist of two data sets, that is, the set of genes involved in a cyclic process and the set of noncycle-involved genes recognized in biological experiments. The cycle-involved genes are used to initialize the proposed algorithm, and the noncycle-involved genes are employed to control the false positives. The expression analysis is composed of the spectral estimation technique and the computation of gene expression distance. The underlying approach relies on the assumption that genes expressing similarly with genes of a process of interest are also likely to participate in that process. This assumption is actually exploited to apply the clustering schemes on the microarray measurements in order to partition genes into different functional groups. The proposed algorithm identifies potential cyclic-process-involved genes and guarantees that the verified cycle genes will be included with 100% certainty into the output gene set, and at the same time the verified noncycle-involved genes are removed from the derived set with 100% certainty. Although most of the existing power-spectra-based algorithms can be crafted into the proposed algorithm seamlessly, herein we are using the Lomb-Scargle periodogram due to its simplicity and good performance. The proposed algorithm will also lay a ground for the following cycle pathway research.

## 2. Methods

The proposed algorithm is composed of a spectral density analysis and a gene distance computation based on the time series microarray data. All existing spectral analysis schemes can be incorporated into the proposed algorithm. However, the Lomb-Scargle periodogram is recommended here due to its convenience of implementation and excellent performance for small sample size. The nonparametric Spearman's correlation coefficient is accepted to construct the measure of distance between two genes.

### 2.1. Lomb-Scargle Periodogram and Periodicity Detection

Microarray measurements usually have a large portion of missing data points. Besides, the sampling frequency is tuned to adapt to nonuniformly occurring events. Lomb-Scargle periodogram appears as an excellent candidate for analyzing these irregular data [14].

Given  time-series observations , , where  stands for the time tag, and  denotes the sampled expression of a specific gene, the normalized Lomb-Scargle periodogram at angular frequency  is defined as follows:(1)

where  and  stand for the mean and variance of the sampled data, respectively, and  is defined as follows:(2)

Let  be the greatest common divisor (gcd) for all intervals  (); Eyer and Bartholdi in [15] proved that the highest frequency that should be searched is given by(3)

Based on the obtained power spectral density, each gene is to be classified as either cyclic or noncyclic. The null hypothesis is usually formed to assume that the measurements are generated by a Gaussian noise stochastic process. For the Lomb-Scargle periodogram,  was shown to be exponentially distributed under the null hypothesis [10], a result which was also exploited in [11]. However, recently Schwarzenberg-Czerny reported in [16] that a beta distribution is more appropriate for small sample size frameworks and the -value for detecting the largest peak  is given by(4)

A rejection of the null hypothesis based on a -value threshold implies that the power spectral density contains a frequency with magnitude substantially greater than the average value. This indicates that the time series data contain a periodic signal, and the corresponding gene is cyclic in expression.

In order to prevent the false positives from overwhelming the true positives, the multiple testing correction is performed to control the -value, which is defined as(5)

where  stands for the number of measured genes, and  represents the sorted -values in ascending order. The part being minimized is an estimate of False Discovery Rate (FDR). Given a -value threshold , through which the number of genes to preserve can then be derived as(6)

### 2.2. Gene Distance Measure

A gene is identified to be a cyclic gene if it satisfies either of two conditions: it passes the periodicity test which is performed on the gene expression measurements, or it is within a small distance from the verified cyclic-process-involved genes. Various distance metrics have been proposed in the clustering literature to capture the distance between genes. These include Pearson's correlation, Euclidean distance, city block distance, mutual information. Because the biological samples are generally highly corrupted and the rank statistics tests, as nonparametric methods, usually behave better when extreme observation exists, we accept here Spearman's correlation coefficient as the core of our distance measure. This distance is obtained for two genes  and  between their expressions across all the available experiments as follows:(7)

where () stand for the rank pair of the measurements of genes  and . The parameter  counts the number of sampling points where both gene  and gene  present available observations. This distance measure always assumes values between 0 and 1.

### 2.3. Algorithm Formulation

The proposed algorithm is formulated as Algorithm 1. Lines 1 to 9 accept inputs and initialize the target cyclic gene set with the spectral analysis results and the prior cycle-involved genes. Inside them lines 4 to 8 exclude genes whose peak periodicity, , is in contrast with the prior knowledge of the frequency range  of the researched phenomenon. Lines 10 to 17 represent the iterative accumulation part. They iteratively insert into the potential cyclic gene set the genes expressed similarly as the genes within that set. Lines 18 to 25 stand for the false positive control part, which constructs the control set iteratively to suppress the potential false positives by using the prior knowledge. Line 26 subtracts the control set from the established target set and finalizes the cyclic gene set. The simulation results on the yeast data set showed that the iterative accumulation part controls the false positives pretty well.

**Algorithm 1:**Identifying cyclic process involved genes.

  1: Input gene expression measurements, all sampled genes (referred as ),

* * experimentally verified cycle-involved genes (denoted as *G*),

* * noncycle-involved genes (represented as *F*) and priori frequency range

* *;

  2: Perform power spectral analysis on gene expression data;

  3: Perform statistical tests so that the periodically expressed genes are

* * recognized and stored in set *C*;

  4: **for***each***do**

  5: * ***if****then**

  6: *  *

  7: * ***end**

  8: **end**

  9: , , specify the distance threshold *t*;

10: **repeat***          *

11: * *;

12: * ***for***each*, **do**

13: *  ***if****then**

14: *   *

15: *  ***end**

16: * ***end**

17: **until**

18: **repeat***          *

19: * *

20: * ***for***each*, **do**

21: *  ***if****then**

22: *   *

23: *  ***end**

24: * ***end**

25: **until**

26: 

27: Output *G*;

The algorithm will surely converge to a set. This is because in each iteration of the accumulation and false positive control part, there have to be new members added into the target gene sets. The number of set members keeps increasing, and the set in the previous iteration is a subset of the later set. However, this increase is upper-bounded by the full gene set that contains all the measured genes. Therefore, both the iterative accumulation part and false positive control part converge, and the proposed algorithm also converges.

Usually some general idea about the phenomenon of interest can be used to determine the two bounds  and  of the frequency range. For example, the circadian rhythm has a periodicity around 24 hours, which can be somehow compressed or prolonged by experimental protocols. If no prior knowledge exists, the set  can be used. The other two thresholds are to be specified. The first is the threshold for the periodicity test. To effectively control the false alarm rate, multiple testing correction can be applied and a -value threshold  can be specified. In practice,  can be chosen around 0.15. This threshold can also be decided by comparing the spectral analysis results with the prior knowledge. Such an approach is more attractive when the proposed algorithm is combined with other periodicity detection methods. We are inclined to use a more stringent threshold, which also represents a trade-off between the number of conserved genes and the number of experimentally verified genes. The second threshold is the distance threshold . It keeps decreasing along the iteration. For example, the initial value is assigned to be 0.25, which means high correlation according to Cohen's rule of thumb [17]. Each iteration decreases this threshold by 0.05 until it reaches 0.1, then it remains constant at 0.1. This technique in practice helps to prevent the amplification of false positives.

## 3. Results

The proposed algorithm was applied on the data sets provided by unicellular *Saccharomyces cerevisiae* (budding yeast) and multicellular *Drosophila melanogaster* (fruit fly), respectively. The *in silico* results are discussed briefly here. The full list of identified potential cell cycle genes is presented in the additional files.

### 3.1. Case Study 1: *Saccharomyces Cerevisiae*

Although various time series data sets have been available, including the experiments on human cells [2], the yeast data set published by Spellman et al. [1] is still among the most popular research targets or benchmarks of computational biology, since this data set excels in its large size of samples and the simplicity of the genome. The mRNA concentrations of nearly 6200 Open Reading Frames (ORF) were measured for the yeast strains synchronized by using four different methods, that is,  factor, cdc15, cdc28, and elutriation. The data set contained in total 73 sampling points for all genes, while there existed missing observations for some experiments. The detected periodicity matched the yeast cell cycle. Our prior knowledge was derived from two sources: Spellman et al. [1] revised 104 cell cycle genes that were verified in previous biological experiments, while de Lichtenberg et al. [18] summarized 105 genes that were not involved in the cell cycle.

**Figure 1:**
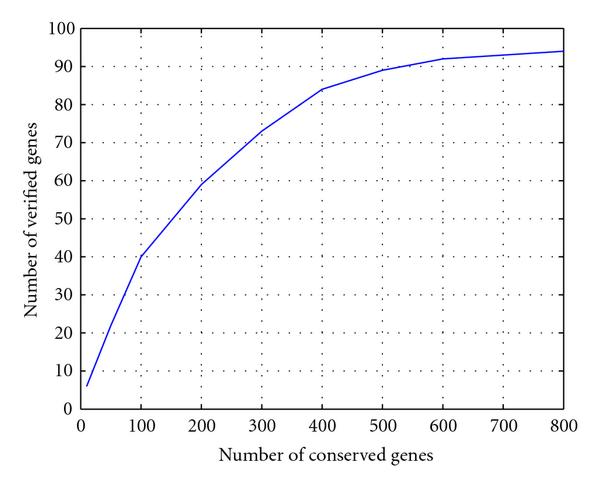
**Performance of Spellman et al.'s CDC score on *Saccharomyces cerevisiae* data**. A specified number of genes are conserved as periodically expressed genes. These genes are compared with the published 104 cell cycle involved genes. The matched genes are counted. Most experimentally discovered cell cycle genes possess high periodicity scores. When the number of conserved genes is greater than 400, Spellman et al. method's identification ability degenerates, as shown by the flat tail of the curve.

Spellman et al. [1] designed a periodicity metric, namely, CDC score, based on the Fast Fourier Transform (FFT) of three experiments  factor, cdc15, and cdc28. The observations of elutriation were discarded due to a computation obstacle. Although later a bunch of other methods were proposed to identify the cell cycle genes, for example, [3, 6, 7], de Lichtenberg found that Spellman's FFT-based method still excelled in testing power and detected the most verified cell cycle genes [8]. However, as admitted in [1], the selection of the number of conserved genes was fairly arbitrary. As Figure 1 illustrates, when the number of conserved genes increases, the number of verified genes increases at a decreasing rate. Actually, after 400 genes have been identified, the curve becomes relatively flat. Therefore, we conserved the 400 genes with top CDC scores as the initialization set in the proposed algorithm. This means a more stringent test threshold for the spectral analysis part.

**Figure 2:**
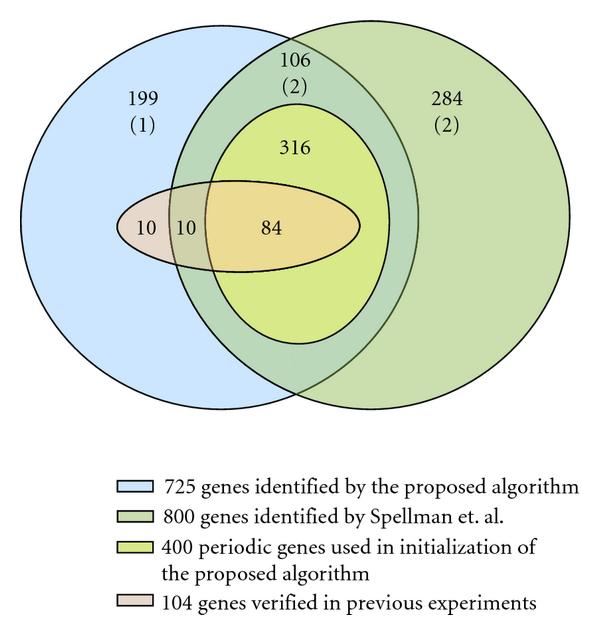
**Venn graph of identified *Saccharomyces cerevisiae* genes**. The proposed algorithm identified 722 genes as potential cell cycle genes. 725 genes were identified before running the false positive control procedure. False positive control removed 3 genes, which are marked within the parenthesis. Various sets are differentiated by their colors.

Figure 2 compares the simulation results with the 800 genes identified by Spellman et al. [1]. Before the running of the false positive control, the proposed algorithm identified 725 genes, in which 104 genes were from the prior experimental knowledge, and 400 genes were from Spellman et al.'s spectral analysis method. These two sets overlapped in 84 genes. We identified 199 genes that were neither identified by Spellman et al.'s method nor reported in the prior knowledge of the 104 genes. The false positive control removed 3 genes and left 722 genes marked as potential cell cycle involved genes. The identified genes are provided in the additional files in MS Excel format.

**Figure 3:**
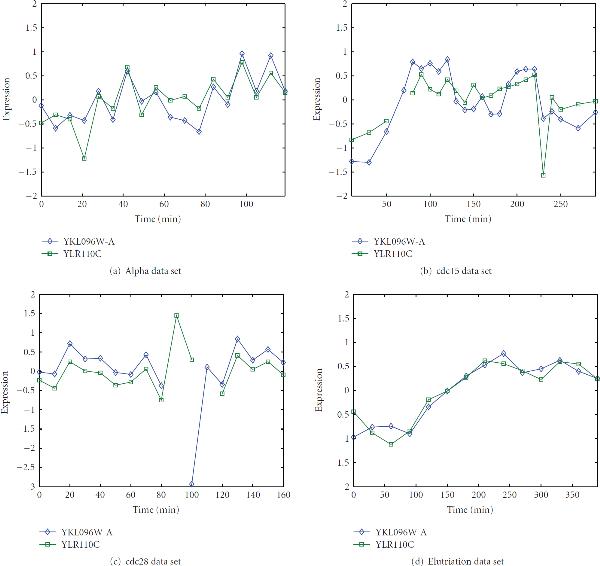
**YKL096W-A(CWP1) and YLR110C(CCW12) time series expressions in four datasets**. Both CWP1 and CCW12 are cell wall protein encoding genes. CWP1 has been verified to be involved in the cell cycle experiment.Alpha data setcdc15 data setcdc28 data setElutriation data set

As an example of a gene detected by the proposed algorithm, Figures 3(a)–3(d) plot time series data for two genes CWP2 (YKL096W-A) and CCW12 (YLR110C). These two genes indicated a strong correlation, with the correlation coefficient 0.19, in their expressions for all four experiments. Both genes are annotated to encode cell wall mannoprotein. CWP2 is cell-cycle regulated at the S/G2 phase [19]. It was assigned a CDC score of 2.031, which ranked 478 in all ORFs. Therefore, it was selected in Spellman et al.'s 800 genes. A stringent CDC score threshold, for example, 2.37 that conserves 400 genes, will make CWP2 discarded from cell cycle genes. CCW12 was not selected in Spellman et al.'s 800 genes because its CDC score was 0.297, which was very low and ranked 4092 in all genes. It has been found that the cell wall accounts for around 30% of the cell dry weight, and its construction tightly coordinated with the cell cycle [20]. Smits et al. [21] summarized that among 43 discovered cell wall protein encoding genes, in which CCW12 was not included at that time, more than half of them were verified to be cell-cycle regulated. In other words, cell wall proteins are highly likely to be involved in the cell proliferation process. Based on the similarity between the expressions of CWP2 and CCW12 in the cell cycle arrest experiments, we hypothesize that CCW12 is also cell cycle regulated at phase S/G2.

**Figure 4:**
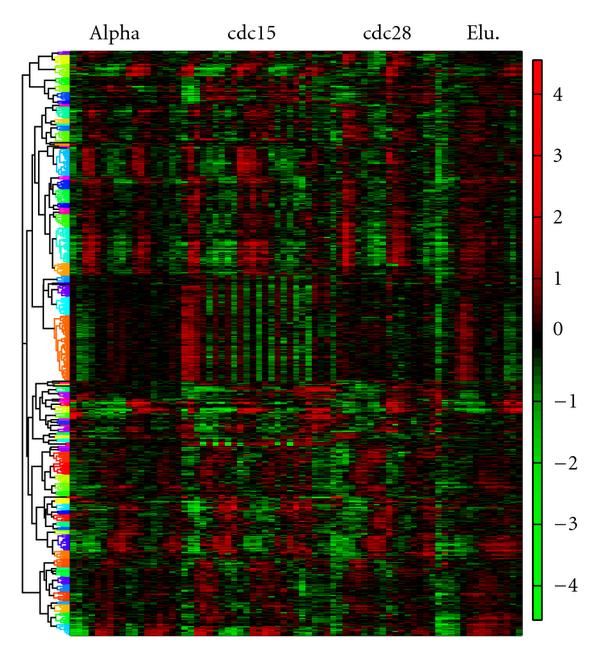
**Clustering analysis of identified *Saccharomyces cerevisiae* genes**. Gene expression levels are indicated by the heatmap. There are 722 genes identified by the proposed algorithm to participate in the cell cycle. Most genes exhibit strong periodicity, as indicated by alternately positioned red and green regions.

**Figure 5:**
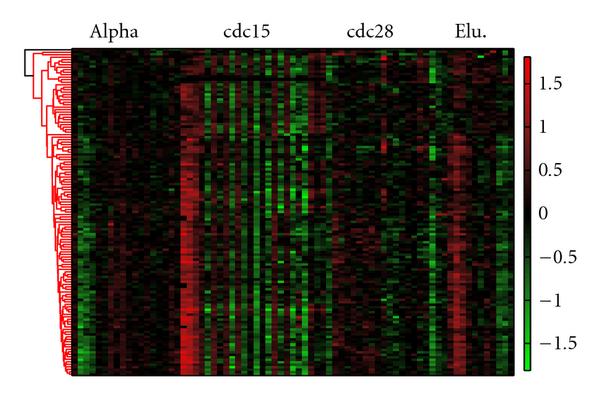
**The exotic clustering of identified Saccharomyces Cerevisiae genes**. Gene expression levels are indicated by the heatmap. This cluster contains 130 genes. The gene expressions in the cdc15 experiment oscillate between low and high levels. Most of these genes are nucleolar genes.

All the detected 722 genes are hierarchically clustered in Figure 4. The hierarchical clustering was selected mainly because it was convenient for visualization, and it avoided to specify the number of desired clusters. It is worthy to note that more advanced methods, for example, self organizing map (SOM) [22] could achieve a better clustering performance. Most clusters indicate a strong periodicity pattern, as can be discerned by the red and green regions which are positioned alternately. There is an exotic cluster, which exhibits fast oscillation in the cdc15 experiments. This cluster contains 130 genes that are illustrated in Figure 5. By examining the existing annotations for these genes, we found that most of them either encode nucleolar proteins or are involved in ribosome biogenesis. It has been verified that ribosome biogenesis consumes up to 80% of proliferating energy, and it is linked to cell cycle in metazoan cells. However, in yeast, the ribosome biogenesis is not regulated by the cell cycle in the same manner as in advanced organisms due to the closed mitosis of the yeast [23]. Defects in nucleolar genes halt the cell at the Start checkpoint [24]. The ribosome biogenesis controls the growth of the size and inhibits the cell cycle until the cell has reached a satisfiable size [25].

In order to measure valid time series samples, the cell culture has to be synchronized. In other words, all cells within the culture should be homogeneous in all aspects, for example, cell size, DNA, RNA, protein, and other cellular contents. Cooper in [26, 27] argued that the ideal synchronization is an impossible mission because different dimensions, like cell size and DNA content, could not be controlled at the same time. Therefore, current popular synchronization methods, like serum starvation and thymidine blocking, are only one-dimensional synchronization methods and fail to achieve a complete synchronization. It is fully possible that the discovered periodicity is completely caused by chance or by the specific synchronization method. Based on the Spellman et al.'s spectral analysis with CDC scores, it is obvious that the most experimentally verified cell cycle genes exhibit top CDC scores. Hence, the spectral analysis is still highly valuable. However, due to the loss of synchronization and nonstationarity, the choice of threshold for the periodicity test has to be much more stringent in order to suppress false positives. When the cell culture is not ideally synchronized or stationary, the spectral analysis may fail for some data sets, such as the elutriation data set. However, the proposed algorithm is still capable to identify a set of genes which are closely correlated to the verified cell cycle genes based on all the available data. The exploitation of the prior knowledge, consisting of experimentally verified cell cycle genes and noncell-cycle genes, can help to improve the detection accuracy and combat the negative effects induced by the loss of synchronization and nonstationarity.

### 3.2. Case Study 2: *Drosophila Melanogaster*

The multicellular *Drosophila melanogaster* serves as a good prototype for the research of mammalian diseases because it has only 4 pairs of chromosomes, on which are located abundant genes with mammalian analogs. Our *in silico* experiments are performed on the *Drosophila melanogaster* data set published by Arbeitman et al. [28]. With the usage of cDNA microarrays, the RNA expression levels of 4028 genes were measured, and these stood for about one-third of all found fruit fly genes. The synchronization of the cell culture was yielded by the Cryonics method. In Arbeitman et al.'s experiments, 75 sequential sampling points were observed, starting right after fertilization and through embryonic, larval, pupal, and early days of adulthood. There were 134 experimentally verified cycling circadian genes [29]. Among these 134 genes, 52 were measured in Arbeitman's experiment [28]. We did not locate the set of noncell-cycle genes in the Drosophila literature. Therefore, the false positive control procedure was not performed. The least time interval between any two sampling points was 30 minutes, which was much larger than the Drosophila's cell cycle period. However, the pupal data set had sufficient sampling points to provide insights into the circadian rhythm.

**Figure 6:**
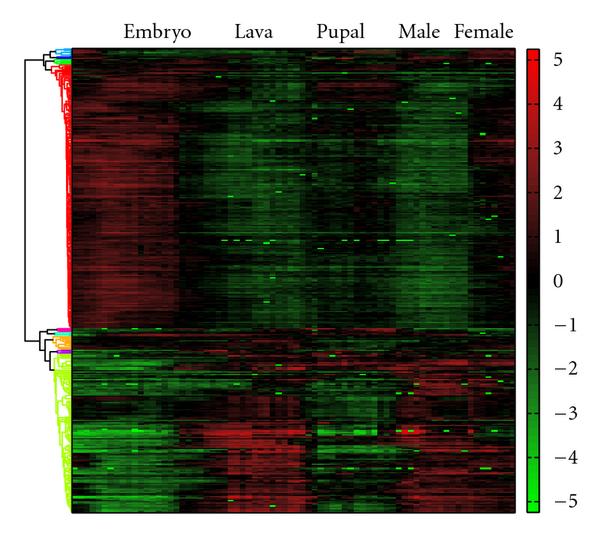
**Clustering analysis of identified *Drosophila melanogaster* genes**. Gene expression levels are indicated by the heatmap. There are 344 genes identified by the proposed algorithm to be involved in the circadian rhythm. The dendrogram can be split into the top and bottom groups, respectively, which are complementary in their expressions.

The spectral analysis was accomplished by applying the Lomb-Scargle periodogram on the nonuniformly sampled pupal data. We found that cyclic genes concentrated most of the power spectral density at the frequency band with the period of tens of hours. By posing a *q*-value threshold at 0.1, 50 genes were preserved for the initialization of the proposed algorithm. Then, there were 344 genes identified by the proposed algorithm. A dendrogram for these genes is illustrated in Figure 6. The top and bottom parts constitute two complementary groups. Most of the experimentally verified genes (46 out of 52) are located in the bottom part, exhibit a transition from the repressed level to the induced level around the time of 11 hours after fertilization.

Two most extensively studied genes involved in the Drosophila circadian rhythm are  and . In Arbeitman's experiment,  showed relatively prominent periodicity in the pupal stage. However, the period was prolonged to be more than 24 hours. This was due to the fact that the synchronization method slowed down the biological process. Unfortunately,  was not measured in the experiment. A large portion of identified genes have been verified to participate in metabolism, a process closely controlled by circadian rhythm. A cross-species knowledge might be valuable. However, special precautions must be considered when the two organisms are too different, like the yeast and fly. The yeast is a unicellular organism with closed mitosis while fly is multi-cellular with open mitosis. The difference between multicellular organisms is less prominent. Therefore, we hypothesize that the prior knowledge of the Drosophila might be valuable for the identification of more advanced species, for example, Homosapiens. The complete list of identified genes is provided in the supplementary materials [30].

## 4. Conclusions

A novel algorithm is proposed to identify the cyclic-process-involved genes through the incorporation of microarray data analysis with the prior knowledge of genes participating in the cyclic process. The *in silico* experiments were conducted based on the data sets corresponding to the unicellular *Saccharomyces cerevisiae* and the multicellular *Drosophila melanogaster*. The potential cell cycle and circadian rhythmic genes were identified and compared with the existing computational results. It is corroborated that the proposed algorithm is capable to exploit all the available data and propose potential cycle-involved genes.
